# Ethical challenges in pathogen sequencing: a systematic scoping review

**DOI:** 10.12688/wellcomeopenres.15806.1

**Published:** 2020-06-03

**Authors:** Stephanie Johnson, Michael Parker

**Affiliations:** 1 Wellcome Centre for Ethics and Humanities and Ethox Centre, University of Oxford, Oxford, OX3 7LF, UK

**Keywords:** pathogen genomics, ethics, global health, infectious disease, NGS

## Abstract

**Background**: Going forward, the routine implementation of genomic surveillance activities and outbreak investigation is to be expected. We sought to systematically identify the emerging ethical challenges; and to systematically assess the gaps in ethical frameworks or thinking and identify where further work is needed to solve practical challenges.

**Methods**: We systematically searched indexed academic literature from PubMed, Google Scholar, and Web of Science from 2000 to April 2019 for peer-reviewed articles that substantively engaged in discussion of ethical issues in the use of pathogen genome sequencing technologies for diagnostic, surveillance and outbreak investigation.

**Results**: 28 articles were identified; nine United States, five United Kingdom, five The Netherlands, three Canada, two Switzerland, one Australia, two South Africa, and one Italy. Eight articles were specifically about the use of sequencing in HIV. Eleven were not specific to a particular disease. Results were organized into four themes: tensions between public and private interests; difficulties with translation from research to clinical and public health practice; the importance of community trust and support; equity and global partnerships; and the importance of context.

**Conclusion**: While pathogen sequencing has the potential to be transformative for public health, there are a number of key ethical issues that must be addressed, particularly around the conditions of use for pathogen sequence data. Ethical standards should be informed by public values, and further empirical work investigating stakeholders’ views are required. Development in the field should also be under-pinned by a strong commitment to values of justice, in particular global health equity.

## Introduction

Genetic information derived from pathogens is an increasingly essential input for infectious disease control, public health and research ^1^. Although routine sequencing of pathogens was, until recently, unthinkable, the Centers for Disease Control (CDC), Food and Drug Administration (FDA), state, and global public health laboratories now routinely sequence more than 200 foodborne bacterial isolates a day and more than 6,000 influenza virus genomes a year ^2, 3^. In the United Kingdom, Public Health England now engages in routine clinical genomic diagnostics and drug sensitivity testing for *Mycobacterium tuberculosis* ^4^. In the research setting, phylogenetic analysis (the study of evolutionary relationships among pathogens) is being used to track and understand factors associated with the spread of infections such as HIV ^5^ and to monitor the global spread of drug-resistant infections ^6^. Mobile genomic sequencing technology is also being applied to disease outbreak investigation, most publicly in the case of the Ebola outbreak in West Africa ^7– 9^. Going forward, the routine implementation of genomic surveillance activities and outbreak investigation is to be expected.

While the technical developments of sequencing technology are being implemented at a rapid pace, the non-technical aspects of implementing this technology are still being broadly discussed between the different stakeholders involved ^1^. The successful implementation of this rapidly developing technology will, for example, require sharing of samples and metadata, interdisciplinary global collaborative partnerships, and will need to offer useful evidence for public health decision-making. Importantly, the successful and appropriate response to these challenges will also require the systematic identification, analysis and addressing of a number of complex ethical, legal and social issues. A number of factors will contribute to the types of ethical issues that arise in different instances. These are likely to include characteristics of the disease, the environmental, political and geographical context, existing laws and policies, public attitudes, and cultural differences ^10^. In the work reported in this paper, taking these and other ethical issues as our focus, we sought to systematically examine the available literature to: identify the emerging ethical challenges and proposed solutions; and to systematically assess the gaps in ethical frameworks or thinking and identify where further work is needed to solve practical challenges.

## Methods

Scoping reviews seek to identify literature relevant to a research objective and may include a variety of research formats and conceptual literature ^11– 13^. This study sought to review published literature on ethical aspects of pathogen sequencing. Inclusion criteria for the study encompassed a broad range of article types, including empirical studies, news articles, opinion pieces, features, editorials, reports of practice, and theoretical articles.

### Search strategy

We systematically searched indexed academic literature from PubMed, Google Scholar, and Web of Science from 2000 to April 2019 for peer-reviewed articles that substantively engaged in discussion of ethical issues in the use of pathogen genome sequencing technologies for diagnostic, surveillance and outbreak investigation. The search was then updated in January 2020. The initial search strategies were developed through an iterative process and used a combination of controlled vocabulary (MeSH terms) and free text words. An example MEDLINE search strategy is provided in Table 1. Reference lists of included articles were searched for relevant articles and further database searchers were conducted using the names of researchers commonly publishing in this field. Finally, we also reviewed relevant international research and clinical practice guidance for relevant guidelines e.g. website of the World Health Organization.

**Table 1.** Medline search strategy.

Genomics	Infectious disease	Ethics
1. genomics [Mesh] *Genomics/es [Ethics]	11 Bacterial Infections/ge, tm [Genetics, Transmission] bacterial	23. Ethics Committees, Clinical/ or Ethics, Research/ or Ethics/ or Ethics, Medical/ or Ethics Committees, Research/ or ethic*.mp.
2. sequencing [tiab]	12. infectious disease	24. Ethic*
3 . Sequence Analysis, DNA [MESH]	13. viral	25. bioethics
4. DNA, Bacterial [MESH]	14. pathogen.mp	26. Genomics/es [Ethics]
5. genom*	15. infectious disease.mp. or Communicable Diseases/	27. policy
6. sequenc*	16. outbreak.mp. or Disease Outbreaks/	28. Guide line
7. Phylogeny [MESH]	17. Population Surveillance/ or Public Health Surveillance/ or surveillance.mp.	29. framework
8. DNA, Viral/ or viral.mp. or Genome, Viral/ DNA, Bacterial/ or Sequence Analysis, DNA/ or sequenc*.mp.	18. epidemic*.mp.	30. Socio-cultural
9. DNA, Bacterial/ or Sequence Analysis, DNA/ or sequenc*.mp. or Phylogeny/	19. epidemic*.mp.	30. 22 or 21 or 22 or 23 or 24 or 25 or 26 or 27 or 28 or 29
10. 1 or 2 or 3 or 4 or 5 or 6 or 7 or 8 or 9	20. Epidemiology/ or epidemiology. mp. or Molecular Epidemiology/	31. 10 and 22 and 31
	21. public health	
	22. 11 or 12 or 13 or 14 or 15 or 16 or 17 or 18 or 19 or 20 or 21	

### Selection criteria

We sought to maximize the literature included in the review by reviewing guidelines, frameworks, commentaries and original research reviews related to pathogen sequencing. We also included studies on molecular typing where enough accuracy could be included to include transmission tracking, as this was thought to provide useful insights into the ethical challenges pathogen sequencing technologies may pose. We excluded studies considering genomics outside of infectious disease or focusing on host response studies as these were not deemed relevant or specific enough to the topic under investigation.

### Selection of studies

Duplicates were removed. SJ undertook title and abstract screening to remove obviously irrelevant studies, borderline cases were discussed with MP and a decision reached by consensus. Data was then abstracted by SJ and cross-checked for accuracy by MP. Names of study authors, institutions, journals of publication and results were non-blinded.

### Analysis

SJ initially inductively analysed all studies, recording the aims and main findings in Microsoft Excel 2016 (16.0.4699.1000), and developing descriptive codes to chart the broad themes describing the literature base. Similar findings were then grouped according to topic area and a preliminary list of themes was developed in collaboration with MP. Both authors engaged in iterative discussions about organization of findings, after which the final themes were decided. SJ subsequently re-examined each study and extracted data using a standard format (design, results and recommendations).

## Results

The search produced 466 articles after duplicates were removed. All articles were initially screened by title and abstract and thirty-nine full text articles were assessed for eligibility. Twenty eight articles were included in the final analysis; three ethical guidelines or frameworks ^14– 17^; seven empirical research studies ^1, 18– 23^; eight reviews of the ethics ^2, 17– 21^; and ten publications that contained a section on the ethical aspects of pathogen sequencing ^24– 33^. The literature largely originated from the US and other high-income countries (HICs, country determined by lead author institution): nine from the United States (US), five from the United Kingdom (UK), five from The Netherlands, three from Canada, two from Switzerland, one from Australia, and one from Italy. Only two publications, by the same author, originated from the global south (South Africa). Eight articles were specifically about the use of sequencing in HIV ^14, 15, 17, 20, 22, 28, 34, 35^. Eleven were not specific to a particular disease. Table 2 presents a summary of studies.

**Table 2.** Summary of studies.

Authors, Year, Country	Year	Country	Disease
**Empirical studies**			
Davies, A., Scott, S., Badger, S, Török, M. E.,& Peacock, S.	2015	United Kingdom	Tuberculosis
Degeling C, Johnson J, Gilbert GL.	2019	Australia	Flu, MRSA and Listeria
Ribeiro Dos, Santos., Martine Y. van Roode , George B. Haringhuizen, Marion P. Koopmans, Eric Claassen, & Burgwal 2018	2018	The Netherlands	Non specific
Mutenherwa F, Wassenaar DR, de Oliveira T. 2018	2018	South Africa	HIV
Rump, C. Woonink, F. Van Steenbergen, J. Verweij, M. Hulscher, M	2017	The Netherlands	Food borne Pathogens
Schairer C, Mehta SR, Vinterbo SA, Hoenigl M, Kalichman M, Little S	2017	United States	HIV
Shean, R, & Greninger, A	2018	Switzerland	Non specific
**Ethics frameworks or guidelines**			
Coltart CEM, Simwinga M, Eba P, Grabowski MK, Amon JJ, Baggaley R, *et al.*	2018	United Kingdom	HIV
David Evans, Nannette Benbow	2017	United States	HIV
World Health Organisation	2018	Switzerland	Non specific
**Reviews of the ethical aspects**			
Greninger AL.	2019	United States	Non specific
Gilbert M, Swenson L, Unger D, Scheim A, Grace D.	2016	Canada	HIV
Johnson SB, & Parker M.	2019	United Kingdom	Non specific
Mehta, S. R., Vinterbo, S. A., Little, S. J.	2014	United States	HIV
Mutenherwa F, Wassenaar DR, de Oliveira T.	2019	South Africa	HIV
Ribeiro, Koopmans & Haringhuizen	2018	The Netherlands	Non specific
Rump, B., Cornelis, C. Woonink, F. &Verweij, M.	2013	The Netherlands	Non specific
Rump, B. & Woonink, F	2012	The Netherlands	Hepatitis A, Group A Streptococcus
**Contained a section on ethics**			
Bhattacharya S	2014	United Kingdom	Hepatitis C and HIV
Boccia S, Pasquarella C, Colotto M, Barchitta M, Quattrocchi A, Agodi A.	2015	Italy	Hospital Acquired Infections
Gardy JL, Loman NJ.	2018	Canada and United Kingdom	Non specific
German D, Grabowski MK, Beyrer C.	2017	United States	HIV
Grubaugh, Nathan D	2019	United States	Non specific
Gwinn M, MacCannell DR, Khabbaz RF.	2017	United States	Non specific
PHG Foundation	2015	United Kingdom	Non specific
Thaler DS, Head MG, Horsley A.	2019	Switzerland	Non specific
Thorogood A, Zawati MH, Knoppers BM.	2014	Canada	Non specific
Yozwiak NL, Schaffner SF, Sabeti PC.	2015	United States	Ebola
**Empirical studies**			
Davies, A., Scott, S., Badger, S, Török, M. E. & Peacock, S.	2015	United Kingdom	Tuberculosis
Degeling C, Johnson J, Gilbert GL.	2019	Australia	Flu, MRSA and Listeria
Ribeiro Dos, Santos., Martine Y. van Roode, George B. Haringhuizen, Marion P. Koopmans, Eric Claassen, & Burgwal	2018	The Netherlands	Non-specific
Mutenherwa F, Wassenaar DR, de Oliveira T.	2018	South Africa	HIV
Rump, C. Woonink, F. Van Steenbergen, J. Verweij, M. Hulscher, M	2017	The Netherlands	Food borne Pathogens
Schairer C, Mehta SR, Vinterbo SA, Hoenigl M, Kalichman M, Little S	2017	United States	HIV
Shean, R, & Greninger, A	2018	Switzerland	Non-specific
**Ethics frameworks or guidelines**			
Coltart CEM, Simwinga M, Eba P, Grabowski MK, Amon JJ, Baggaley R, *et al.*	2018	United Kingdom	HIV
David Evans, Nannette Benbow	2017	United States	HIV
World Health Organisation	2018	Switzerland	Non-specific
**Reviews of the ethical aspects**			
Greninger AL.	2019	United States	Non-specific
Gilbert M, Swenson L, Unger D, Scheim A, Grace D.	2016	Canada	HIV
Johnson SB, & Parker M.	2019	United Kingdom	Non-specific
Mehta, S. R., Vinterbo, S. A., Little, S. J.	2014	United States	HIV
Mutenherwa F, Wassenaar DR, de Oliveira T.	2019	South Africa	HIV
Ribeiro, Koopmans & Haringhuizen	2018	The Netherlands	Non-specific
Rump, B., Cornelis, C. Woonink, F. &Verweij, M.	2013	The Netherlands	Non-specific
Rump, B. & Woonink, F	2012	The Netherlands	Hepatitis A, Group A Streptococcus
**Contained a section on ethics**			
Bhattacharya S	2014	United Kingdom	Hepatitis C and HIV
Boccia S, Pasquarella C, Colotto M, Barchitta M, Quattrocchi A, Agodi A.	2015	Italy	Hospital Acquired Infections
Gardy JL, Loman NJ.	2018	Canada and United Kingdom	Non-specific
German D, Grabowski MK, Beyrer C.	2017	United States	HIV
Grubaugh, Nathan D	2019	United States	Non-specific
Gwinn M, MacCannell DR, Khabbaz RF.	2017	United States	Non-specific
PHG Foundation	2015	United Kingdom	Non-specific
Thaler DS, Head MG, Horsley A.	2019	Switzerland	Non-specific
Thorogood A, Zawati MH, Knoppers BM.	2014	Canada	Non-specific
Yozwiak NL, Schaffner SF, Sabeti PC.	2015	United States	Ebola

Results were organized into four themes: Tensions between public and private interests; difficulties with translation from research to clinical and public health practice; the importance of community trust and support; equity and global partnerships; and the importance of context.

## Theme 1: Tensions between private and public interests

When considering the implications of collecting, using and sharing pathogen genome sequence data, the interests and rights of individuals were universally acknowledged. In particular, the literature pointed to the importance of considering and/or protecting individual rights to autonomy ^19, 21, 32, 36^, and to privacy ^18, 21, 22, 28^. Many pointed out that the potential of sequencing techniques to detect the origin and routes of transmission of an outbreak may result in negative consequences for the individuals involved ^25^. Consequences may include stigmatization, penalties, economic risks, problems with interpersonal relationships (e.g. inadvertent disclosure of infidelity), emotional distress and the capacity for discrimination ^19, 21, 22, 24, 25, 27, 30, 32, 37– 39^. There was also concern that sequencing could lead to serious legal consequences, particularly with regards to the criminalization of HIV transmission ^14, 15, 17, 22, 24, 28, 34, 35^. It was also acknowledged that individuals may have an interest in avoiding the use of information about them for purposes they do not endorse, such as to support anti-gay sentiment or as part of a criminal investigation ^20, 24^. Some were concerned about forced testing either of certain groups such as gay men ^28^ or healthcare workers ^19, 21, 38– 40^. There was acknowledgment of individual professional interests of researchers and practitioners to ownership and use of data ^1, 16, 32^.

Sequencing was also seen to carry risks for communities and groups. Many authors noted that certain groups can be placed at risk through characterization as high risk or likely to transmit virus (HIV), including geographically defined groups, sexual or gender minorities, or those defined by ethnicity, nationality, or migration status ^14^. Similarly, data regarding transmission patterns of multidrug-resistant tuberculosis (TB) could be used for discrimination based on ethnicity, and possible challenges to immigration ^20^. Institutions could be subject to increasing numbers of legal claims, or companies could suffer reputational or economic damage ^39^. It was also noted that some communities may be particularly at risk of being exploited by research, especially during emergency outbreak situations ^14, 32^.

On the other hand, it was acknowledged that widespread availability and use of sequence data contributes important benefits to the clinical and research communities ^17^. In particular, the rapid sharing of data can help identify etiological factors, predict disease spread, evaluate existing and novel treatments, symptomatic care and preventive measures, and guide the deployment of limited resources ^16^. Many of the ethical, legal and social issues arising from sequencing studies were seen to reflect this tension between interests that arise at the level of the individual and these other important societal interests (in public health) ^14, 16– 19, 21, 22, 27, 28, 32, 33, 37, 39^. Much of the literature either explicitly or implicitly reflected on how to balance these tensions. Usually, by reflecting on the permissibility of conducting sequencing studies, and on the necessary conditions of collection, storage and use of data.

### Permissibility of sequencing studies and conditions of use

There was strong support for the permissibility of conducting sequencing studies, as long as potential risks were thoughtfully mitigated ^14, 18, 19, 21, 22^. In one empirical study, patients and healthcare workers were asked if the benefits of HIV molecular epidemiology outweigh the risks; all said yes ^22^. Three-quarters of respondents answered with an unqualified, yes, and one quarter gave a positive answer with qualifications, such as ‘It’s very necessary, just as long as parameters are set in place and they’re kept’, or ‘with proper protections in place, the benefits outweigh the risks’ ^22^. In another study, expert Delphi panelists held that the protection of the public was of overriding importance, but that most of the potential harms could be managed ^19^.

There were differences across the literature in the priority afforded to different conditions of use, and to the types of risk or amount of risk deemed acceptable. It was broadly agreed that any research should have a favourable risk benefit ratio ^14, 20^, and that maximizing the utility of data must be weighed against concerns over interests of individuals and that policies on data collection and release should seek to align the interests of different parties ^36^. There was, however, disagreement as to whether privacy concerns or public interest should take precedence ^24, 25, 31^ and some noted that the balance between the public health benefit and personal privacy risk for individuals whose genetic data (personal or pathogen) are included is difficult to delineate, since neither the true benefit nor the actual risk to participants has been adequately defined ^27, 35^.

Below we set out the key recommendations from the literature on the conditions of use for pathogen sequence data. Box 1 summarises recommendations from the literature for future research focus and study design.

Box 1. **Recommendations from the literature for future research focus and study design**

**Social science and ethics research**

1. Community engagement should occur early in the research design process, ensuring that phylogenetic research is relevant to participating communities and that local perspectives are included in the design and overall conduct of research studies ^14^.
2. Preparation for future outbreaks should include provisions for rapidly building new bridges and establishing community norms ^32^.
3. Future research on the perceptions of stakeholders should include standardised background information ^35^.
4. Social and behavioural research into conceptual and normative aspects should be backed up by empirical research ^35^.
5. New inter-disciplinary collaborations including microbiologists, engineers and bioethicists ^30^.
6. As real-time and other intervention strategies that build on HIV phylogenetic information continue to emerge, it will be critical to address questions of efficacy for cluster growth interventions to ensure that the benefits outweigh potential risks. Implementation science research may also inform best practices for discussing the meaning and limitations of sequence data and cluster membership with community members and help to identify acceptable and evidence-based approaches that impose the least risk to persons within specific contexts. These might involve partnerships with providers for non-intrusive patient follow-up related to clusters, more detailed consent procedures for future follow-up related to HIV test results or partner services referrals, and specific guidelines and education to mitigate criminalization risks ^28^.
7. Communication methods that increase the understanding of phylogenetic studies need to be designed and evaluated. These must emphasise potential harms, thoughtful mitigation of harms to risk groups, processes for monitoring risk, and clear protection procedures to minimise risks ^14^.
8. Collaboration between stakeholders is necessary, with an active exchange of experiences and best practices. The first step, should be sought in creating awareness and consensus within sectors on the causal factors of barriers to sharing of sequence data ^1^.

**Ethical conduct of studies**

9. Need to pre-define exceptional circumstances where un-validated techniques might be used in emergency situations ^27^.
10. To ensure scientific validity, researchers and their associates should be competent to implement the proposed study design. In order to maximize scientific validity, the researchers should ensure that they have all necessary resources, that the community accepts the protocol and that a competent and independent research ethics committee (REC) or institutional review board (IRB) reviews and approves the protocol ^35^.
11. The scientific objectives of research should guide the choice of participants and determine the inclusion criteria and appropriate recruitment strategies. It is unethical to use privilege, convenience and/or vulnerability as criteria for selecting participants. Exclusion of certain population sub- groups or communities in a research study without appropriate scientific justification is also considered unethical ^35^.
12. Risk mitigation strategies must also provide for redress mechanisms in cases of abuse or misuse of phylogenetic data. These strategies might require the establishment of ties with local legal services, organisations working to protect people with HIV, and criminalised or stigmatised populations, to ensure that they have access to the means to protect their rights ^14^.

### Publication

It was clear that the release of information of relevance to public health should not be delayed by publication timelines or concerns over academic ownership of data ^16, 26, 32^. Recommendations to address such conflicts of interests included: that medical journals should update their policies to support pre-publication sharing of pathogen sequence data related to outbreaks ^16^; publication disclaimers prohibiting use of sequence data for publication without permission ^16, 32^; acknowledgment of data sharing contributions and the inclusion of such criteria to the assessment of academic research credit ^1^; establishment of governance structures and dispute resolution mechanisms that can mediate where disagreements arise ^16^.

### Anonymization and privacy protection

Much of the literature suggested that traditional methods of de-identification or anonymization of data are insufficient to meet their purpose in the context of pathogen sequencing ^2, 14, 17, 23, 28, 33, 39^. Existing approaches to minimize the risk of privacy loss to participants are based on de-identification of data by removal of a predefined set of identifiers ^17^. However, this has three key limitations in the context of pathogen genomics. First, sharing of corresponding sample metadata (minimally time and place of collection, ideally with demographic, laboratory, and clinical data) is essential to enhance the interpretation and the value of genomic data ^16^, therefore removal of key identifiers such as geographic location may severely limit the utility of genomic data ^39^. Second, removing pre-defined identifiers may be ineffective at protecting privacy and confidentiality ^17, 19, 22, 39^. For example, one study demonstrated how sample collection dates associated with microbiological testing at a large tertiary hospital were highly correlated with patient admission date (protected health information), meaning data is re-identifiable ^23^. Small study populations may also mean that individuals who are part of a transmission chain may be able to identify others during the course of routine contact tracing (e.g. sexual partners) ^17^. Third, anonymization of data does little to mitigate potential risk to communities and groups ^14, 17, 39^. Perhaps a consequence there was clear support for re-visioning of existing privacy standards, and for privacy policies specific to the context of sequencing studies.

### Consent

There was debate in the literature around the importance of consent to the use of sequence data and associated meta-data in epidemiological investigation. In the research setting, Coltart *et al.* ^14^ state that research participants and patients whose samples are being used for phylogenetic analysis should ideally have consented to such use, but suggest that when using data from previous studies, where only broad consent for HIV-related research might have been obtained, waivers of specific consent are allowable when samples are no longer linked to identifiers, or when broad consent for sample collection for research and storage in future studies was given ^14^. In the public health and clinical setting, an Australian study reported that one of the key differences amongst participants in a modified Delphi study included the necessity for consent before testing and data-linkage. No panelists agreed with the statement “under no conditions should a study be conducted without prior consent”, although only ten of thirty agreed that consent is not required under any conditions ^21^. In a Dutch study, outbreak managers thought intervention without seeking explicit consent of all individuals involved is justified when there is at least be a substantial public health threat, realistic expectation that deploying the techniques will help to mitigate the outbreak, and that source and contact tracing would most likely not be successful without the use of molecular typing techniques ^21^.

### Data access

There was a strong commitment to rapid and open data-sharing, particularly in emergency or outbreak situations ^16, 27, 32^ and in such conditions for incentives and safeguards to encourage rapid and unrestricted access to data release ^16, 27, 32^. The World Health Organization (WHO) recommended that in emergency outbreak situations “the first set of sequences providing crucial information on the pathogen, genotype, lineage, and strain(s) causing the outbreak should be generated and shared as rapidly as possible. Sharing of corresponding anonymised sample metadata (minimally time and place of collection, ideally with demographic, laboratory, and clinical data) is essential to enhance the interpretation and the value of genomic data” ^16^. However, access to data gathered as part of clinical care was seen as ethically more contentious as “publicly accessible databases are not an appropriate storage location for the level of metadata required to enable clinical and epidemiological analysis for the purposes of providing patient and population care” ^39^. Suggestions were made for a tiered approach to data release, whereby a separate database governed by appropriate public health authorities would collate and store metadata in a location to which access to data could be limited to users with a legitimate clinical or public health need to use it, and data that cannot be released into public domains but is needed by authorised healthcare and public health professionals for service delivery remains within a suitable secured access database ^39^.

A public survey on TB explored questions related to database access and the potential benefits and risks associated with it. Most felt that medical professionals and the research community should have access to such a database; and a significant proportion thought that other agencies, such as the police (10%) and immigration officials (13%), should also have access to the genomic database. Experts, however, were clear that they felt transmission data should not be used in litigation; this was partially because it was deemed too unreliable ^24, 27^ and also because of the potential for ‘abuse’ of data ^14, 15, 24^.

Overall, there was broad support for further work in defining the conditions for collection use and storage of data and samples ^2, 27, 30, 32, 37^ and for policy and legal clarity to aid the ethical implementation of these technologies. This will require more work to carefully assess and understand risks ^39^; research to decide how much individual privacy might be risk in the name of public health ^31^; consideration of alternative strategies required to mitigate this risk, such as suppression of data in the public domain where it may cause serious harm; and adjustments to communication plans ^14^.

## Themes 2: Difficulties of translation from research to clinical and public health practice

Effective phylogenetic work often occurs at the interface between research and public health practice because the same data can be used for both purposes ^14^. In this regard, pathogen sequencing was described as ‘straddling the boundary between research and clinical use’ ^27^. The hybrid nature of sequencing activities imposes important ethical challenges.

### Genomics and the clinic

At present, sequencing technology results are produced with a substantial delay from sampling; therefore, any results are unlikely to be timely in informing clinical care ^14, 39^. However, with the evolution of real-time phylogenetic and other typing techniques, reporting of results to study participants could result in changes to clinical management ^14^. This could present a number of new problems for clinical practice. For example, whether HIV acquisition events are linked to the known infected partner might be crucial for interpretation of the efficacy of prevention strategies, but feeding back individual results that indicate source of HIV acquisition in discordant couples would require ethical reflection ^14^. Likewise, routine surveillance could impact on clinical and public health encounters. In one empirical study interviewees pointed out:

“So, what would it mean? At some point we knock on someone’s door and say, “from the data we have it looks like you are infecting a lot of people and we need you to stop doing that.” This then becomes an issue in the public health setting and it seems you cannot do that in a research study if people can now perceive you can knock at individual doors and say you have a problem here, then people might be very reluctant to participate in further research studies and might be very reluctant to provide information to public health professionals because of the fear that they could be singled out” ^20^.

Clinical implementation of metagenomics sequencing (un-targeted testing) has the potential to detect unexpected or incidental findings that may include infections with hepatitis or HIV ^39^. Incidental findings of a different type may occur if non-germline samples (such as faecal samples) are contaminated with germline cells, which could potentially reveal predictive information about developing inherited disease ^39^. Furthermore, informed consent for phylogenetic studies that are difficult complex and difficult to understand, and in which the benefits and risk may not be fully determined, may also be difficult to achieve ^35^. Mutenherwa suggests that where sequence data are generated for routine clinical management, its subsequent use for research and surveillance may be underestimated by patients ^20^. Others suggested that the right to withdraw from research activities—a key indicator of voluntary participation in research—was overlooked by expert stakeholders ^20^.

### Professional boundaries

Understanding and interpreting phylogenetic data requires significant expertise ^25, 27^ and presents a challenge to established professional boundaries. Expertise in phylogenetic studies creates new obligations for researchers, such as deciding whether or not to participate in forensic investigations and potential prosecutions of individuals ^24^, and to consider the down-stream uses and misuses of data ^14^. The routine implementation of pathogen sequencing studies may create new responsibilities for clinical microbiologists (related to public health) ^2^, and require major changes in culture such that diagnostic interpretation, therapeutic management decisions and antimicrobial treatment regimes are delegated to physicians instead of microbiologists ^31^.

## Theme 3: Truth-telling, trust and community engagement

Many noted that there are important reasons to ensure that the public and individuals understand the uses of data collected as part of a sequencing studies, and the potential risks. First, this was seen to have some intrinsic value in that it supports patient autonomy and truth telling is a respected moral virtue ^14, 15, 19, 21^. Second, truth-telling was seen to be important because it may lead to better outcomes in research and public health practice. This is both because this was deemed to promote trust in research and therefore lead to increased participation, and because it promotes disclosure, which is helpful from a public health perspective. Third, promoting understanding of uses and risks of data was also seen as a way of avoiding harm and exploitation of vulnerable individuals and communities, by enhancing understanding of risks that may be specific to that them. In some cases, this was balanced by a number of practical challenges to telling people the truth, such as: risk of fear mongering ^19^; information needed for legal proceedings in public interest ^14^; and the fact that it may be difficult to adequately inform the public and/or ensure full understanding ^20^. None-the-less, there was a clear recommendation in the literature to raise public awareness and understanding of these techniques ^19, 22, 30^, and for early and meaningful community engagement ^15, 22, 32, 34^ prior to conducting sequencing studies. This was seen as particularly important when working with vulnerable groups or when the risks of participation are high.

## Theme 4: Justice and global partnerships

The notion of justice appeared to be a widely recognized ethical principle in the field ^1^. Stakeholders pointed to the importance of equitable access to data ^27^, and to benefit-sharing obligations ^16, 26^. This included an ethical imperative that outbreak related research and countermeasures, such as diagnostics and vaccines, should be accessible to all affected countries ^26^, and towards reciprocal arrangements such that countries that participate in sequencing activities should derive some corresponding local benefit ^27^.

### Collaboration

Collaboration between researchers from Africa and HICs was raised as an important ethical consideration ^20^. In one empirical study, interviewees were concerned that African researchers were not meaningfully engaged in the scientific research process in health research in general and phylogenetic research in particular, and that for equitable and mutually beneficial collaborative research partnerships to be realized, local researchers were encouraged to take leading and active roles throughout the research process ^20^. This type of collaborative research practice was supported elsewhere in the literature ^14, 27, 37^. For some, this was to enhance equity as well as to help maximize the utility of data and lead to better public health outcomes. It was argued that local researchers were more likely to understand their health care and research systems and study results were more likely to be easily translated into policy ^20^, and that context specific responses to particular outbreaks were likely to be required. Recommendations were made to conduct studies exploring the nature of existing collaborative partnerships between researchers from low- and middle-income countries (LMICS) and HICs to explore team composition and distribution of roles, including contribution to intellectual property ^20^. Outbreak related research and countermeasures, such as diagnostics and vaccines, must be accessible to all affected countries not only as a legal obligation, but also as an ethical imperative ^26^.

### Global cooperation

It was also noted that global and interdisciplinary partnerships are a necessary component of an effective genomic informed response to infectious disease. This was because of the vast range of stakeholders and varying interests involved in control of infectious disease outbreaks, and because issues may resist simple resolution and span multiple jurisdictions ^27^. For example, conflict may result from governments wishing to keep an outbreak quiet and/or from the tension between LMICs with few resources for generating and using data and the researchers or response teams from better-resourced settings ^1, 37^.

Ownership of samples and data was seen as an important barrier to global cooperation. The Nagoya Protocol (NP), for example, was developed to facilitate access to genetic resources and the fair and equitable sharing of benefits arising from their utilization ^1^. Nevertheless, despite the importance of reinforcing sovereignty rights of States over genetic resources in their territory, uncertainties about intellectual property rights and the resulting disputes hamper access to samples ^1, 32^. Ribeiro *et al.* ^1^ explain that:

“The real or perceived possibilities for the commercial valorization of microbial genetic resources (MGR) has enforced their appropriation for further use in research, innovation and product development. The problem for public health surveillance occurs when such appropriation is triggered at initial (upstream) phases of the research and innovation cycle, such as sampling and sequencing of microorganisms, instead of later stages, such as the actual product development (in this case drugs, diagnostics and vaccines). As such, stakeholders are reluctant to share their (intangible) assets even in early phases of the innovation process, decreasing the scope of innovation efforts due to the lack of access to upstream research inputs.”

The same authors suggest that standardized and simplified sharing agreements ^26^, and collaboration between stakeholders with an active exchange of experiences and best practices ^1^ are required.

In general, recommendations were made for a global approach to ethics, policy and legal frameworks ^16, 27, 29, 32, 37^. For example, it was suggested global data sharing arrangements should include “a global data governance or ethical framework, supplemented by local memoranda of understanding that take into account the local context” ^29^; or to investigate how the Global Alliance for Genomics and Health (GA4GH) framework for responsible data-sharing could be adapted for digital pathogen surveillance ^27^.

## Themes 5: The importance of context

Lastly, it was clear that the types of ethical issues likely to arise are in part dependent upon the contexts in which studies are conducted, as well as the nature of the pathogen under study. Chiefly, information that may impact on interpersonal relationships was viewed as particularly sensitive and therefore, worthy of additional ethical reflection. Examples included: sexually transmitted infections ^14, 20^; consent requirements to use isolates collected from dead neonates for the purposes of epidemiological research ^19^; and disclosure of family members as the source of infection ^38^. It was suggested that the balance of risks to patients and public health benefits is likely to be affected by the characteristics of the pathogen, in terms of likely morbidity and mortality: infectivity; treatability and drug resistance ^39^. Stakeholders also suggested that the ethical permissibility of sharing data about, particularly with regards to the source of transmission, may be different in professional contexts, where healthcare providers or companies are seen to carry a responsibility to control risk, as opposed to outside of professional contexts where protecting individuals from ‘naming and shaming’ may be of greater concern ^21^. It was also noted that the legal and regulatory structures in which studies are conducted may also influence the implementation and ethics of conducting pathogen sequencing studies. In particular, use of phylogenetic analyses in criminal convictions was raised as an ethical risk ^14, 17, 24^.

## Limitations of the review

Although quality assessment of all included materials is desirable in systematic reviews, it was not possible in this case due to the inclusion of a diverse range of research formats and literature, such as commentaries and ethics guidelines. A second limitation of this review is that a large proportion of the literature included related to phylogenetic and HIV specifically (8 out of 28), meaning that the issues relevant to this context may be over-represented.

## Conclusion

This review highlights that while pathogen sequencing has the potential to be transformative for public health and clinical practice and to bring about important health benefits, there are a number of key ethical issues that must be addressed. In particular, there was clear support in the literature for innovative and critical thinking around the conditions of use for pathogen sequence data. This includes context specific standards of practice for consent, data collection, use and sharing. These practices should be informed by public values, and further empirical work investigating stakeholders’ views are required. This should include experts in pathogen sequencing, patients and the general public, as well as end users such as public health professionals and clinicians. Lastly, it is both a scientific and an ethical imperative that development in the field is under-pinned by a strong commitment to values of justice, in particular global health equity.

## Data availability

All data underlying the results are available as part of the article and no additional source data are required.
