# Supplementary material for: Ethical challenges in pathogen sequencing: a systematic scoping review
Source: Wellcome Open Res. 2020 Jun 3;5:119. [Version 1] doi: 10.12688/wellcomeopenres.15806.1 (PMC7445679; doi:10.12688/wellcomeopenres.15806.1)
Supplement: Peer review history

# Peer review: Reviewer response for version 1

**DOI:** 10.21956/wellcomeopenres.17333.r39902

**Published:** 2020-08-24

**Authors:** Yann Joly

**Affiliations:** 1 Center for Genomics and Policy (CGP), McGill University, Montreal, Canada

This manuscript presents the results of a scoping review of the literature on the topic of the ethical issues in the use of pathogen genome sequencing technologies. The authors make excellent use of the scoping review methodology which they implemented clearly (good use of tables!) and rigorously.

 There are minor formatting issues and typos:

 p. 4 under theme 1: **Somewere** concerned about forced testing either of certain groups such as gay men ^28^ or healthcare workers ^19,21,38–40^. There was acknowledgment of individual professional interests of **researchersand** practitioners to ownership and use of data.

 p. 9 under theme 3: ‘that may be specific to **that** them’

 The main substantial issue is the substantial focus of the manuscript on the HIV context (8 articles out of 28) which the authors acknowledge. HIV is a particularly stigmatized, serious condition, with lifelong consequences for patients which is not the case for many other communicable diseases. The problem is that in the past few months, COVID-19 has been a game changer in the field. COVID-19 is both much more contagious but less stigmatizing and, for most people, less dangerous than HIV. Considering that the last update to this scoping review was made in January 2020, none of the COVID-19 emerging literature was considered. The result, which is not the fault of the authors, is that the article will only have a limited relevance to the current global pandemic. Given the importance of COVID to the field and beyond, it could be worth it for the author to take the time to update their research accounting for very recent developments before publishing. Otherwise, the publication maybe perceived as already outdated and not garner much attention from readers.

 A second issue of the manuscript is that while it acknowledges the tension and blurry demarcation between the research and the public health context, it doesn’t really provide any solution in this regard. For example, in the context of pathogen genome sequencing for outbreak surveillance during a public health emergency, informed consent is often not required. However, the lack of consent can create issue later for data sharing with the research community. Such a scenario is not really discussed in the manuscript. Similarly, the impact of the public health vs. research situation on the potential requirement for ethics review is not discussed. Perhaps this was not touched upon in the literature, but it is certainly a preoccupation of researchers in the COVID context.

Is the work clearly and accurately presented and does it cite the current literature?

Partly

If applicable, is the statistical analysis and its interpretation appropriate?

Yes

Are all the source data underlying the results available to ensure full reproducibility?

Yes

Is the study design appropriate and is the work technically sound?

Yes

Are the conclusions drawn adequately supported by the results?

Yes

Are sufficient details of methods and analysis provided to allow replication by others?

Yes

Reviewer Expertise:

Ethical, legal and social issues of genomic research, medical law, bioethics.

I confirm that I have read this submission and believe that I have an appropriate level of expertise to confirm that it is of an acceptable scientific standard.

# Peer review: Reviewer response for version 1

**DOI:** 10.21956/wellcomeopenres.17333.r39365

**Published:** 2020-07-06

**Authors:** Gregory L. Armstrong

**Affiliations:** 1 Office of Advanced Molecular Detection, National Center for Emerging and Zoonotic Infectious Diseases, Centers for Disease Control and Prevention, Atlanta, GA, USA

The article is generally well written, although it would be stronger if it had a more concise, more focused set of recommendations for readers to act on. The focus of the paper is mostly on the use of microbial genomic data for research but also touches a little on the issues around public health use of the data. This reviewer is not an expert in ethics; that said, the ethical concepts brought out in the paper in the review appear to be appropriate and informative. Some additional concepts that weren’t included, possibly because the article presents a review of already published ideas, are the following:

- The potential for civil legal consequences from publication of genomic data. The article discusses at a few different points the potential for criminal prosecution, but publication of data could also open up participants of a study to civil penalties as well. 
- The potential for violation of a privacy when cases are rare. For example, early in an outbreak, as is highlighted in the article, there is an urgency to making sequence data from the pathogen in question public. However, it’s common early in an outbreak for the media to discover and publish the names of early cases. In that situation (which is relatively common), there’s a risk that sequence data made public could be linked to a specific person.
- One concern that is often under-appreciated is that, under certain circumstances, a researcher or public health agency might be legally compelled to release the identity of someone. In general, public health laws are quite strong in shielding public health data, but that may not be the case with research data. If there is uncertainty about whether such data are protected, any participants in research should be notified.
- GH4GE is mentioned in the manuscript, but there’s no mention of PHA4GE ( https://pha4ge.github.io/), which is much more applicable here.
- Issues around the Nagoya Protocol are addressed, but the tension between GISAID and INSDC is not. GISAID provides protections for intellectual property rights that the INSDC members do not, and such protections are key for participation of LMICs. However, where public funds are used to obtain data, there is a strong argument that the data should be made publicly available (i.e., through INSDC) without restriction as long as privacy and confidentiality are not placed at risk. The tension between the two models has been particularly strong with the advent of COVID-19, and the very assertive push by GISAID to prevent researchers from submitting to INSDC or from citing it.

Some (minor) specific issues that need to be addressed:

- I would remove the reference to Microsoft Excel. There’s a strong argument for including information about statistical software, but which spreadsheet is used is not particularly relevant.
- “Theme 1: …”: There are typos in the first paragraph.
- Box 1, Item 5: This is not a complete, declarative sentence like the others.
- Reference 29 appears to be the incorrect reference. Also, the quote from it (Pages 9-10) appears to be a quote from a separate document (that should be cited separately, if still available).

Is the work clearly and accurately presented and does it cite the current literature?

Yes

If applicable, is the statistical analysis and its interpretation appropriate?

Not applicable

Are all the source data underlying the results available to ensure full reproducibility?

No source data required

Is the study design appropriate and is the work technically sound?

Yes

Are the conclusions drawn adequately supported by the results?

Yes

Are sufficient details of methods and analysis provided to allow replication by others?

Yes

Reviewer Expertise:

Pathogen genomics to support public health.

I confirm that I have read this submission and believe that I have an appropriate level of expertise to confirm that it is of an acceptable scientific standard.
